# Insulin-Like Growth Factor Binding Protein 6 Is Secreted in Extracellular Vesicles upon Hyperthermia and Oxidative Stress in Dendritic Cells But Not in Monocytes

**DOI:** 10.3390/ijms21124428

**Published:** 2020-06-22

**Authors:** Massimo Conese, Lorenzo Pace, Nicoletta Pignataro, Lucia Catucci, Antonio Ambrosi, Sante Di Gioia, Nicola Tartaglia, Arcangelo Liso

**Affiliations:** 1 Department of Medical and Surgical Sciences, University of Foggia, Via Napoli 121, 71122 Foggia, Italy; massimo.conese@unifg.it (M.C.); lorenzo.pace@unifg.it (L.P.); nicoletta.pignataro23@gmail.com (N.P.); antonio.ambrosi@unifg.it (A.A.); sante.digioia@unifg.it (S.D.G.); nicola.tartaglia@unifg.it (N.T.); 2 Department of Chemistry, University of Bari, Via Orabona 4, 70126 Bari, Italy; lucia.catucci@uniba.it

**Keywords:** insulin-like growth factor binding protein 6, dendritic cells, monocytes, hyperthermia, microvesicles, exosomes, oxidative stress

## Abstract

Recently, insulin-like growth factor binding protein 6 (IGFBP-6) has been shown to play a putative role in the immune system, as monocyte-derived dendritic cells (Mo-DCs) are stimulated by hyperthermia to express IGFBP-6 at both the mRNA and protein levels. However, the presence of IGFBP-6 in extracellular vesicles (EVs) and whether other pro-inflammatory stimuli can induce IGFBP-6 expression in Mo-DCs are not known yet. In this brief report, we show that hyperthermia (39 °C) induces IGFBP-6 secretion associated with microvesicles and exosomes as early as 3 h. Moreover, free IGFBP-6 is found in conditioned media (CM) of hyperthermia- and H_2_O_2_-treated Mo-DCs, but not in CM obtained from monocytes similarly treated. These results show that diverse inflammatory stimuli can induce IGFBP-6 association with EVs and secretion in conditioned medium, indicating a role for IGFBP-6 in communication between immune cells.

## 1. Introduction

Insulin-like growth factor (IGF) binding protein 6 (IGFBP-6) regulates the action of IGFs, but it is also involved in processes independent of IGFs. As a regulator of IGFs, the main function of IGFBP-6 is inhibiting IGF-II-mediated actions, such as cell proliferation, differentiation, migration, and survival in many cell lines [1]. Such activities are complemented and integrated by the effects given by IGFBP-6 independently on IGF-II, including regulation of proliferation, apoptosis, angiogenesis, and cell migration [2,3]. Recently, a role of IGFBP-6 in inflammatory reactions and diseases has been delineated [4]. We have shown that IGFBP-6 has chemoattractant properties toward cells of the innate (neutrophils, monocytes) and adaptive (T cells) immunity [5,6]. This effect may be relevant in autoimmune disorders for the regulation of the inflammatory microenvironment. Indeed, IGFBP-6 was found at higher levels in sera and sinovial tissue of rheumatoid arthritis patients than in samples derived from patients with osteoarthritis [7]. Interestingly, we have shown that IGFBP-6 is induced in monocyte-derived dendritic cells (Mo-DCs) by hyperthermia (39 °C) at the level of mRNA and protein level, specifically at earlier (3 h) and later (48 h) times, respectively [5]. It is not known whether other pro-inflammatory stimuli can determine a regulation in IGFBP-6 mRNA and protein levels. Cytofluorimetric analysis indicated that intracellular-found staining was lowered at 3 h, but the protein could be not found in the conditioned medium, suggesting that IGFBP-6 revelation was either hampered by dilution factors or confinement in vesicles. Extracellular vesicles (EVs) can be broadly classified into three main categories [8,9]: (1) Microvesicles/microparticles/ectosomes, 50–1000 nm in size, which are produced by outward budding and fission of the plasma membrane; (2) exosomes, ranging from 30 to 150 nm in diameter, which are formed within the endosomal network and released upon fusion of multi-vesicular bodies with the plasma membrane; (3) apoptotic bodies, which are released as blebs from cells undergoing apoptosis. EVs may have a role in both the origin and progression of the acquired immune response, acting at different levels and on different cells [10]. For example, DCs and other antigen-presenting cells secrete EVs (microvesicles and exosomes) containing peptide–MHC I or II complexes and co-stimulatory molecules, which can contribute to antigen presentation to T cells [11].

In this brief report, we have investigated if IGFBP-6 is associated with extracellular vesicles and established the role of oxidative stress in the induction of IGFBP-6 protein expression in Mo-DCs.

## 2. Results

### 2.1. IGFBP-6 Is Found Associated with Microvesicles and Exosomes from DCs upon Hyperthermia Exposure

Previously, we determined that the conditioned medium of monocyte-derived DCs contained IGFBP-6 upon challenge with hyperthermia (39 °C) but only after 48 h of incubation [6]. On the other hand, cytofluorimetric analysis showed that intracellular IGFBP-6 levels decreased at shorter times (3–8 h), suggesting that it was secreted. We reasoned that either this amount of IGFBP-6 was too low for detection by ELISA or that it was contained in extracellular vesicles (EVs) and thus not free to be detected. In order to establish whether IGFBP-6 was present in EVs, we prepared both microvesicles (MVs) and exosomes (EXOs) from Mo-DCs challenged with either 37 °C or 39 °C at various time points. As shown in Table 1, MVs and exosomes presented particle size, polidispersity, and zeta-potential compatible with previously published studies. These parameters did not change substantially with time.

**Figure 1:**
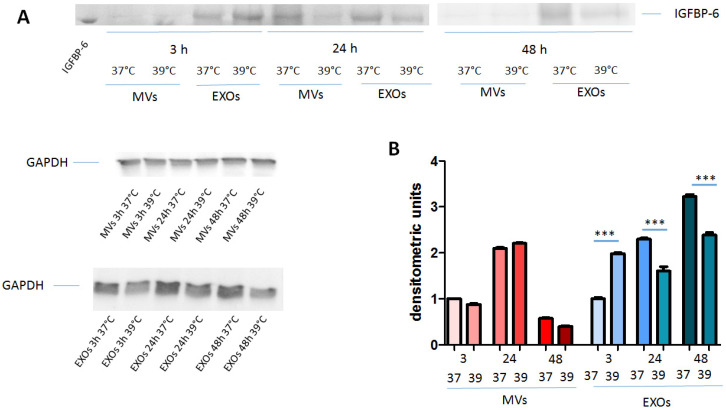
Microvesicles (MVs) and exosomes (EXOs) derived from monocyte-derived dendritic cells (Mo-DCs) contain IGFBP-6. Mo-DCs were incubated at either 37 °C or 39 °C for 3 h, 24 h, or 48 h, and at each time point, MVs and EXOs were isolated from the conditioned medium. (**A**) Western blotting of IGFBP-6 and GAPDH. Purified IGFBP-6 (0.2 μg) was loaded as positive control. (**B**) Densitometric analysis of IGFBP-6 normalized to GAPDH. Intensity of MVs and EXOs IGFBP-6 at 37 °C for 3 h was put to 1 as reference. The results are shown as mean ± SD of two experiments. *** *p* < 0.0001.

Western blotting analysis to detect IGFBP-6 was performed on MV and EXO preparations. MVs and EXOs presented a band specific for IGFBP-6 (25 kDa) that presented variations in intensity according to time (Figure 1A). The densitometric analysis shows that, after normalization to GAPDH (37 kDa), MVs presented a peak of IGFBP-6 protein at 24 h (Figure 1B; *p* < 0.0001, both 3 h and 48 h vs. 24 h), without differences between 37 °C and 39 °C at different time points. Conversely, IGFBP-6 found in EXOs increased steadily at 37 °C from 3 to 48 h (*p* < 0.001, 24 h and 48 h vs. 3 h). At 39 °C, there was also an increase but only at 48 h as compared to 3 h and 24 h. Interestingly, IGFBP-6 protein was significantly higher at 39 °C than at 37 °C after 3 h of incubation (*p* < 0.0001), but was significantly lower at 39 °C than at 37 °C both at 24 h and 48 h (both *p* < 0.0001). Overall, these results show that IGFBP-6 is present in EVs secreted from Mo-DCs and that hyperthermia can modulate its content only in EXOs.

### 2.2. IGFBP-6 Is Secreted by Stimulated DCs

**Figure 2:**
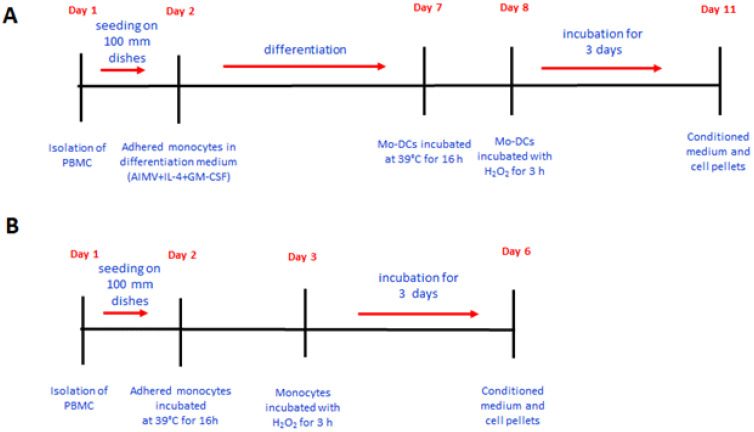
Flow diagram of the experiment. (**A**) Mo-DCs were obtained upon incubation of monocytes with serum-free medium (AIMV) in the presence of IL-4 and GM-CSF starting on Day 2. On Day 7, Mo-DCs were incubated at 39 °C for 16 h, while other dishes were incubated with H_2_O_2_ for 3 h on Day 8. An incubation of 3 days started for all dishes on Day 8 and ended on Day 11. (**B**) The same protocol was used for monocytes, except that they were exposed to hyperthermia on the day after they were plated (Day 2) or incubated with H_2_O_2_ on Day 3. The incubation of all dishes at 37 °C started on Day 3 and finished on Day 6.

To examine whether IGFBP-6 could be found free in the conditioned medium upon hyperthermia, we applied a protocol by which not were only Mo-DCs incubated at 39 °C for 88 h (16 h at 39 °C and 72 h at 37 °C) (Figure 2A) but also the conditioned medium was concentrated 100×. In parallel, Mo-DCs were incubated with H_2_O_2_ for overall 75 h (3 h with the stimulus and 72 h without) (Figure 2A). H_2_O_2_ has already been shown to induce IGBP-6 secretion in a dose-dependent manner [12]. Monocytes were treated in the same manner as Mo-DCs (Figure 2B).

**Figure 3:**
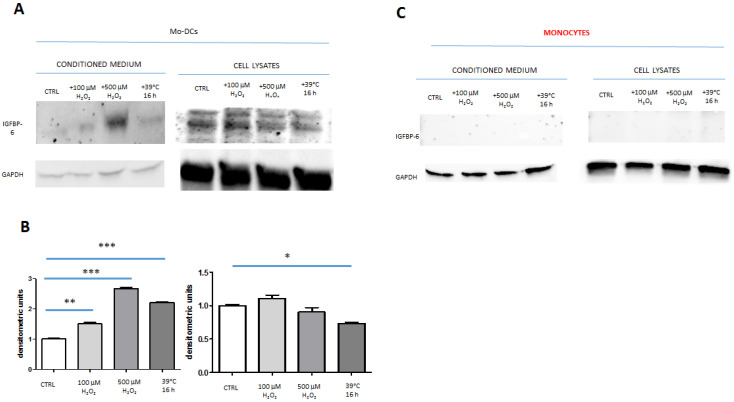
Hyperthermia and H_2_O_2_ stimulate IGFBP-6 secretion by Mo-DCs. (**A**) Western Blotting of IGFBP-6 and GAPDH on conditioned medium and cell lysates of Mo-DCs. (**B**) Densitometric analysis of IGFBP-6 normalized to GAPDH. Intensity of IGFBP-6 at 37 °C for the whole experiment length (CTRL) was put to 1 as reference. The left and right graphs correspond to results obtained with conditioned medium and cell lysates, respectively. The results are shown as mean ± SD of two experiments. (**C**) Western Blotting of IGFBP-6 and GAPDH on conditioned medium and cell lysates of monocytes * *p* < 0.05; ** *p* < 0.01; *** *p* < 0.0001.

Western blotting analysis on conditioned medium revealed that stimulation of Mo-DCs at 39 °C for 16 h and subsequent incubation at 37 °C for a further 3 days brought to IGFBP-6 accumulation with significantly higher levels as compared with control Mo-DCs, i.e., incubated at 37 °C for all the duration of the experiment (Figure 3A; compare first and fourth lanes of the left blot). IGFBP-6 in cell lysates presented two bands, described as glycosylated and non-glycosylated forms [12], which were decreased when Mo-DCs were exposed to hyperthermia (Figure 3A; compare first and fourth lanes of the right blot). The densitometric analysis revealed that hyperthermia exposure increased IGFBP-6 in the conditioned medium by 2.18-fold (*p* < 0.0001), whereas IGFBP-6 decreased in cell lysates by 0.72-fold (*p* < 0.05) (Figure 3B).

Stimulation with H_2_O_2_ for 3 h and further incubation for 3 days at 37 °C also determined the increase of IGFBP-6 in conditioned medium in a dose-dependent manner (Figure 3A; second and third lanes of the left blot), while the IGFBP-6 bands did not show appreciable intensity alterations in cell lysates (Figure 3A; second and third lanes of the right blot). As shown in Figure 3B, the densitometric analysis showed that Mo-DCs treated with hypothermia (37 °C) and then with 100 μM H_2_O_2_ show an increase in the amount of IGFBP-6 found in conditioned medium as compared with hypothermia-only controls (Figure 3B; compare first and second bars of the left graph), similarly to those cells treated with hypothermia and then 500 µM H_2_O_2_ (Figure 3B; compare first and third bars of the left graph). However, while H_2_O_2_ at both doses increased IGFBP-6 secretion in the conditioned medium, the expression of IGBP-6 in cell lysates did not change significantly (Figure 3B; compare second and third bars with the first one in the right graph). Interestingly, monocytes that were stimulated under the same experimental conditions of Mo-DCs did not express any IGFBP-6 protein nor in the conditioned medium or in cell lysates (Figure 3C).

## 3. Discussion

DCs are important cells of the innate immunity that can recognize, phagocytose, and process different types of microrganisms [13]. Moreover, DCs can recognize products released by damaged cells and then contribute to the induction of inflammation also supporting T cell activation [14]. By releasing EVs, DCs can present antigens through them; thus, they can activate both primed and naive T cells, with both activation or tolerogenic effects [10]. DCs have been shown to secrete both microvesicles (with a mean size above 200 nm) and exosomes (with a mean size below 200 nm), and proteomic analysis established that multiple EVs share several intracellular proteins, including GAPDH [15].

Previously, we have shown that Mo-DCs differentially expressed IGFBP-6 mRNA following exposure to 39 °C as compared to monocytes. At the protein level, intracellular staining determined that there was a significant reduction of IGFBP-6 signal at 3 h, while the membrane signal did not change, suggesting that exposure to hyperthermia could decrease the intracellular pool of already synthetized IGFBP-6 [5]. However, no extracellular free IGFBP-6 could be detected at 3 h, but only at 48 h, by an ELISA method based on magnetic beads. We undertook this study to understand whether IGFBP-6 could be found in EVs produced by DCs stimulated by hyperthermia. The evidence that we dealt with EVs congruent with both MVs and EXOs was obtained from physical parameters, such as the size and surface charge. We reasoned that this was the more direct way to assure that we were studying both kinds of EVs, while detection of multiple markers is another possibility to study EVs [8,15]. IGFBP-6 was found associated with both MVs and EXOs, and its levels increased with time, although a different behavior was observed for the protein associated with either EXOs or MVs. The accumulation of proteins inside EVs has been previously described by others [16,17]. In particular, Giusti et al. [16] observed by Western blot that CD63 and HLA levels presented a higher level upon 18 h stimulation as compared with 30 min. Conversely, the total GAPDH signal for the two samples (30 min and 18 h) measured by means of densitometric analysis was equivalent, indicating that this process is selective for EV markers. Interestingly, EXOs were the most secreted EVs at 30 min, while MVs were enriched at 18 h of incubation [16], a behavior resembling the one we noticed with DC-derived EVs, although our experiment extended to 48 h (Figure 1B). This time-dependent process may depend on the different pathways involved in their generation: EXOs derive from the endocytic pathway, while MVs are generated by pinching of the plasma membrane [18]. EXOs results were complementary to those previously obtained with cells [5], as IGFBP-6 was increased at 3 h post-stimulation. Interestingly, at 24 and 48 h, IGFBP-6 decreased at 39 °C as compared with 37 °C, a result compatible with the increase of intracellular staining at these time points [5]. Notably, IGFBP-6 in MVs showed a peak at 24 h but with no differences between 37 °C and 39 °C, indicating that this increase was not due to hyperthermia exposure. It is commonly accepted that MVs and EXOs represent important vehicles of intercellular communication in between cells locally or at a distance. In particular, EXOs are characterized by a defined content of proteins, RNAs, and microRNAs that are released into target cells and have the potential to modulate several pathological scenarios, including the maintenance of tumor microenvironment [19]. In DCs, the role of exosomes in cellular and humoral immune activation is well recognized, while less is known for the role of MVs [20,21,22]. The significance of IGFBP-6 associated with EVs in immune regulation will be the focus of further studies.

Different agents, such as IGFs, cAMP, retinoic acid, vitamin D, 17-estradiol, p53, and glucocorticoids have been shown to induce IGFBP-6 synthesis and secretion in different cells by affecting transcriptional and post-transcriptional mechanisms [3,23,24,25]. So far, the regulation of the production of IGFBP-6 by DCs remains elusive. To get more insight into this, we tried to understand if the lack of IGFBP-6 in CM derived from DCs upon hyperthermia exposure at early times (3 h) [5] was a matter of experimental design. Thus, we applied the protocol published by Xie et al. on fibroblasts [12], who showed that IGFBP-6 was found in concentrated conditioned medium upon stimulation with sub-lethal H_2_O_2_ concentrations. This protocol allowed us to establish that H_2_O_2_ induced effectively the secretion of free IGFBP-6 by Mo-DCs in a dose-concentration manner. Moreover, a 3 h incubation of Mo-DCs at 39 °C (and other 3 days at 37 °C) induced a 2.18-fold increase in IGFBP-6 level in concentrated conditioned medium as compared with cells incubated at 37 °C. However, while hyperthermia induced a significant decrease in IGFBP-6 in cell lysates, albeit not as expected on secreted levels, H_2_O_2_ did not cause any significant change in intracellular IGFBP-6, suggesting that the secreted form of IGFBP-6 under oxidative stress conditions was likely due to new protein synthesis.

These results show that IGFBP-6 secretion is induced by hyperthermia and oxidative stress in Mo-DCs, indicating that IGFBP-6 may play a role in activating T cells in a pro-inflammatory microenvironment. Since only 3 h at 39 °C are needed for this secretion, IGFBP-6 seems to be a sensitive sensor of the inflammatory milieu. This role for IGFBP-6 should be further studied by functional assays involving innate and adaptive immune cells.

The limit of our study is that we have focused on in vitro generated Mo-DCs, while a number of studies have shown that they are not only morphologically different but also functionally different, from the naturally occurring, in vivo circulating myeloid DCs (BDCA-1 and BDCA-3, now called CD11c+ and CD141+, respectively). They differ from Mo-DCs in their ability to stimulate T cells, endocytose particles, immune complexes, and soluble molecules, as well as in cytokine production in response to pathogen signals [26,27,28,29,30]. The evaluation of IGFBP-6-positive EVs derived from circulating myeloid DCs and how these cells respond to different stimuli in secreting IGFBP-6 will validate our results obtained in Mo-DCs and will be the focus of future studies. We also have not studied whether circulating EVs transport IGFBP-6 in vivo in patients with pyrexia, and this clinical study will have to be performed.

Overall, this brief report shows that IGFBP-6 is found both associated with DC EVs and free in conditioned medium upon hyperthermia exposure and that it is secreted upon challenge with oxygen peroxide, a pro-inflammatory stimulus.

## 4. Materials and Methods

### 4.1. Generation of Monocyte-Derived Dendritic Cells

Specific approval of the local ethics committee was obtained for this study (Ospedali Riuniti University Hospital, cod 30/CE/2014, approved on 19 February 2014 day month year). Written informed consent was obtained from all participants. Peripheral blood mononuclear cells (PBMC) from healthy donors were isolated from buffy coats by density centrifugation using a Ficoll-Paque PLUS Lymphocyte separation (d = 1007 g/mL) gradient (GE Healthcare, Rome, Italy), as described by the manufacturer. After density centrifugation, PBMC were plated in 100 mm dishes flasks at a density of 30 × 10^6^ cells in 5 mL of complete medium AIMV (Invitrogen, Frederick, MD, USA) and incubated overnight at 37 °C and 5% CO_2_. The following day, the monocytes were adhered, the medium was changed to eliminate the unadhered cells, and cells were cultured with medium containing GM-CSF (50 ng/mL) (Schering-Plough, Milan, Italy) and IL-4 (1000 U/mL) for 6 days. In parallel experiments, adhered monocytes were used as such.

### 4.2. Isolation and Characterization of Microvesicles and Exosomes

Microvesicles and exosomes were obtained by the differential centrifugation method. Monocyte-derived DCs were incubated either at 37 °C or 39 °C for different time intervals (3, 24, and 48 h) in 100 mm dishes, and at each time point, the conditioned medium was centrifuged at 2000× *g* for 30 min at 4 °C. The supernatant was filtered (0.8 μm) and centrifuged at 12,000× *g* for 45 min at 4 °C. The exosome-containing supernatant was filtered (0.2 μm) and centrifuged at 110,000× *g* for 70 min, while the microvesicles contained in the pellet were resuspended in 800 μL of cold PBS and centrifuged at 12,000× *g* for 45 min. Pellets of exosomes and microvesicles were either resuspended in 50 μL of PBS and processed for physical analysis or resuspended in 30 μL of RIPA buffer containing 25 mM Tris HCl pH 7.4, 150 mM NaCl, 1% (*v*/*v*) NP-40, 1% (*w*/*v*) sodium deoxycholate, 0.1% (*w*/*v*) SDS, and protease inhibitors cocktail (Thermo Scientific, Rodano, Italy, cat n. 87785). Protein content was evaluated by the Bradford method according to the manufacturer’s instructions (Bio-Rad, Hercules, CA, USA). Twenty micrograms of proteins were analyzed by Western blotting. Purified IGFBP-6 (Peprotech, London, UK) was used a positive control (0.2 μg).

The particle size and polydispersity index (PDI) of samples were determined by photon correlation spectroscopy (PCS) using a Zetasizer Nano ZS (Malvern Panalytical Ltd., Malvern, UK). Zeta-potential determination was performed using laser Doppler anemometry (Zetasizer Nano ZS) after dilution in KCl solution (1 mM, pH 7.0). PDI is a number calculated from a two-parameter fit to the correlation data (the cumulants analysis). This index is dimensionless and scaled such that values smaller than 0.05 are mainly seen with highly monodisperse standards, while PDI values bigger than 0.7 indicate that the sample has a very broad particle size distribution.

### 4.3. Analysis of IGFBP-6 Protein in Conditioned Medium and Cell Lysates

DCs and monocytes were treated either with H_2_O_2_ (at 100 and 500 μM) for 3 h or posed in hypertermic condition at 39 °C for 16 h. Conditioned media were collected after 3 days of treatments. The serum-free conditioned media were filtered through a 0.45 μm filter to remove cell debris and concentrated 100 times down in volume using a speed vacuum concentrator. Cells were detached by trypsin/EDTA and centrifuged at 800× *g* for 10 min to obtain cell pellets.

### 4.4. Western Blotting Analysis

Cell pellets were lysed with 100 μL of lysis buffer (20 mM Tris pH 7.5, containing 300 mM saccharose, 60 mM KCl, 1.5 mM NaCl, 5% glycerol, 2 mM EDTA, 1% TritonX-100, 1 mM PMSF, 1 mM disodium orthovanadate, 1 mg/mL leupeptin, and 2.5 mM sodium phyrophospahte). Cell lysates were incubated on ice for 30 min and centrifuged at 12,000 rpm for 30 min. Protein concentration in the concentrated medium and cell lysates was determined by the Bradford method and stored at −20 °C until they were analyzed.

Thirty μg of proteins were separated by SDS-PAGE on precasted 12% polyacrilamide gels (Bio-Rad, cat no. 4568044) under reducing conditions and then transferred to nitrocellulose membranes. Protein markers (Opti-Protein XL Marker; Applied Biological Materials Inc, Richmond, BC, Canada; cat no. G266) were run aside samples on each gel. Membranes were incubated for 60 min at room temperature with Western Blocker Solution (Sigma Aldrich, Milan, Italy, cat. no. W0138) to block aspecific binding and incubated overnight with the primary antibody anti-IGBP-6, washed, and incubated with a goat anti-mouse IgG-HRP Conjugate (Bio-Rad, cat no. 1706516). In parallel, other gels were run with the same samples and incubated with a mouse antibody directed against GAPDH. The following mouse monoclonal antibodies were utilized: anti-human IGFBP-6 (R&D Systems, Minneapolis, MN, USA, cat. no. MAB8761) and anti-human GAPDH (Santa Cruz Biotechnology, Dallas, TX, USA, cat no. Sc-32233). Specific proteins were revealed using the enhanced chemiluminescence (ECL) reagent (Bio-Rad). Images were obtained by ChemiDoc XRS^+^ System (Bio-Rad). Densitometric analysis was carried out by using the software ImageJ and normalized by using the expression of the housekeeping gene GAPDH.

### 4.5. Statistical Analysis

The difference among different treatment groups was analyzed by ANOVA with Tukey’s Multiple Comparison test using GraphPad Software v. 4 (La Jolla, CA, USA). *p* < 0.05 was considered statistically significant.

## Figures and Tables

**Table 1:** Particle size, polydispersity index (PDI), and zeta-potential (Z-potential) of MVs (microvesicles) and EXOs (exosomes) derived from dendritic cells (DCs).

Sample	Dimension(nm)	PDI	Z-Potential (mV)
MVs 37 °C 3 h	369 ± 25	0.32	−12.1 ± 0.4
MVs 39 °C 3 h	294 ± 22	0.38	−12.7 ± 0.3
EXOs 37 °C 3 h	105 ± 7	0.52	−11.0 ± 2.1
EXOs 39 °C 3 h	119 ± 11	0.49	−9.5 ± 1.2
MVs 37 °C 24 h	291 ± 7	0.29	−12.4 ± 0.6
MVs 39 °C 24 h	332 ± 17	0.41	−12.1 ± 0.3
EXOs 37 °C 24 h	94 ± 8	0.34	−10.3 ± 0.8
EXOs 39 °C 24 h	121 ± 13	0.46	9.2 ± 0.4
MVs 37 °C 48 h	298 ± 17	0.28	−12.0 ± 1.0
MVs 39 °C 48 h	272 ± 15	0.36	−11.7 ± 0.4
EXOs 37 °C 48 h	106 ± 13	0.36	−9.5 ± 0.8
EXOs 39 °C 48 h	169 ± 7	0.41	−8.5 ± 0.6

The results are shown as mean ± SD of two experiments, each conducted in triplicate.

## Abbreviations

EXOs	Exosomes
Mo-DCs	Monocyte-derived dendritic cells
MVs	Microvesicles
